# Rainfall’s impact on agricultural production and government poverty reduction efficiency in China

**DOI:** 10.1038/s41598-024-59282-2

**Published:** 2024-04-23

**Authors:** Jianlin Wang, Zhanglan You, Pengfei Song, Zhong Fang

**Affiliations:** 1 School of Finance, Fujian Business University, Min Merchants Research Center, Fuzhou, 350506 People’s Republic of China; 2 School of Business Administration, Fujian Business University, Fuzhou, Fujian 350007 People’s Republic of China; 3 The Center for Economic Research, Shandong University, Jinan, Shandong 250100 People’s Republic of China; 4 School of Economics, Fujian Normal University, Fuzhou, Fujian 350007 People’s Republic of China

**Keywords:** Rainfall, Agricultural production, Government poverty reduction, Sustainability efficiency, Meta-DDF, Environmental economics, Sustainability

## Abstract

The quest to eradicate poverty, central to the United Nations Sustainable Development Goals (SDGs), poses a significant global challenge. Advancement in sustainable rural development is critical to this effort, requiring the seamless integration of environmental, economic, and governmental elements. Previous research often omits the complex interactions among these factors. Addressing this gap, this study evaluates sustainable rural development in China by examining the interconnection between agricultural production and government-led poverty reduction, with annual rainfall considered an influential factor of climate change impacts on these sectors and overall sustainability. Utilizing a Meta-frontier entropy network dynamic Directional Distance Function (DDF) within an exogenous Data Envelopment Analysis (DEA) model, we categorize China’s 27 provinces into southern and northern regions according to the Qinling-Huaihe line for a comparative study of environmental, economic, and governmental efficiency. This innovative approach overcomes the limitations of previous static analyses. The findings reveal: (1) Rainfall, as an exogenous variable, significantly affects agricultural production efficiency. (2) The overall efficiency in both southern and northern regions increases when accounting for rainfall. (3) Government effectiveness in poverty reduction is comparatively lower in the northern region than in the southern region when rainfall is considered. These insights underscore the importance of including climatic variables in sustainable development policies and emphasize the need for region-specific strategies to bolster resilience against climatic challenges.

## Introduction

As it is at the center of the United Nation’s Sustainable Development Goals (SDG), overcoming poverty in all its manifestations continues to be one of the most significant hurdles humanity confronts. Currently, developing countries are faced with the enormous challenges of poverty eradication and agricultural development. As the world’s most populous country, since its reform and opening up, China has lifted millions out of poverty, but progress has been uneven^1^. According to the World Bank^2^, over the past four decades, China has reduced poverty by nearly 800 million people, accounting for more than 75 percent of global poverty reduction over the same period. However, this does not mean that China’s poverty reduction efforts have been completed. Relative poverty will persist in China for a long time, with some regions still facing serious risks and challenges of people returning to poverty after being lifted out of poverty.

The United Nations Development Programme (UNDP)^3^ has emphasized that the emergence of new threats such as the impacts of climate change on rainfall necessitates additional efforts to uplift people from poverty. Climate change and its effects on rainfall patterns pose significant challenges to agricultural systems worldwide, with far-reaching implications for food security and poverty alleviation^4^. The significance of rainfall as a determinant of agricultural productivity is well-established^5^. Studies have consistently shown that agricultural output is profoundly influenced by variations in rainfall patterns. Research by Liu and Feng^6^ and Wang et al.^7^ have delved into the spatial and temporal dimensions of rainfall, emphasizing the need for precision in evaluating its impact on different crops and regions. The efficiency of rainfed agriculture, which is predominant in many parts of China, has become a crucial factor. Studies by Zhang et al.^8^ and Chen et al.^9^ provide insights into the efficiency of rainfed farming systems, exploring adaptive strategies such as improved water management and drought-resistant crop varieties. These findings contribute to a nuanced understanding of how farmers can enhance productivity in the face of changing rainfall patterns.

The nexus between rainfall variability and agricultural production serves as a focal point in understanding the challenges and opportunities facing Chinese farmers. Efficiency evaluations of these poverty reduction policies are critical to ensuring their effectiveness. Studies^10,11^ underscore the spatial and temporal nuances of rainfall patterns, emphasizing the need for adaptive strategies. Efficiency evaluations reveal that technological interventions, such as precision agriculture and data-driven decision-making^12^^,^ play a pivotal role in mitigating the impact of unpredictable rainfall, enhancing resource utilization, and improving crop yields. Research by Chen et al.^9^ delves into the livelihood outcomes of farmers in regions prone to erratic rainfall, providing evidence of the direct linkages between agricultural efficiency, income stability, and poverty reduction. Therefore, the efficiency evaluation of the impact of rainfall on agricultural production and government poverty reduction in China unveils a complex and dynamic landscape. The intricate interplay between climatic factors, agricultural practices, and government policies forms a tapestry that shapes the socio-economic fabric of the nation.

Efficiency evaluations of government poverty reduction policies elucidate the successes and challenges of interventions aimed at improving the livelihoods of vulnerable populations. Research by Li et al.^10^ indicates that rural infrastructure development projects contribute to poverty reduction by enhancing connectivity and creating opportunities. However, challenges persist, and the effectiveness of policies is contingent upon addressing regional disparities, administrative bottlenecks, and ensuring inclusive growth^7^. The efficiency evaluation extends beyond immediate poverty reduction outcomes to encompass the broader context of climate change adaptation and environmental sustainability. Studies^13,14^ underscore the importance of integrating sustainable agricultural practices into poverty reduction strategies. By recognizing the intrinsic link between environmental resilience and poverty alleviation, efficiency evaluations provide insights into long-term strategies that promote both economic development and ecological sustainability.

The main contributions of this study are as follows: (1) From the perspective of sustainable agricultural development, a novel two-stage poverty research theoretical and analytical framework is constructed, divided into agricultural production and government poverty reduction stages, to obtain a more objective efficiency evaluation and better under-stand the current state of agricultural production and poverty reduction in China. (2) Considering public expenditure on central government poverty alleviation funds and local government agricultural support funds as inputs for the second stage, the number of rural residents guaranteed minimum living standards as an unexpected output, and the Sustainable Agricultural Development Infrastructure Index as an expected output, this study comprehensively evaluates the efficiency of China’s agricultural production and poverty reduction. (3) By combining the advantages of the meta-frontier entropy network DEA model, this study creatively improves the model by introducing rainfall as an exogenous variable. In addition, this study divides China’s 27 provinces into northern and southern provinces according to the “Qinling-Huaihe” line for a comparative study of environmental, economic, and governmental efficiency. This allows researchers and policymakers to perform a more detailed analysis of the efforts to eliminate poverty and provides valuable insights for policymakers to pro-mote inter-sectoral collaboration for sustainable agricultural development and optimize the use of public funds.

## Literature review

### Government poverty reduction efficiency

Concerning the government’s role in poverty reduction, a significant number of scholars have dedicated their studies and achieved fruitful results. Two prominent schools of thought, represented by Douglas C. North and Commons, Mitchell, and Veblen, challenge neoclassical economics’ limited consideration of institutions and governance in the economy^15^. While these schools share a common emphasis on the importance of institutions, they differ in their methodologies^16^.

The empirical study’s conclusion, against the above backdrops, reveals contentious debates^17^ and the definition of efficiency in government poverty reduction is still under research. Some results indicate that efficient governance acts as a prerequisite to poverty alleviation in various countries^2,15,18–21^, while some studies argue that the benefits of efficient government are primarily enjoyed by upper-income and middle-income groups, limiting their impact on poverty reduction and reaching the poor^22–24^. Recent research by Christiaensen and Martin^25^ provides nuanced insights into how growth in agriculture, often spurred by government policies, is more poverty-reducing than equivalent growth in non-agricultural sectors. This highlights the need for targeted government policies that specifically address the needs of the poor, especially in rural areas where agriculture is a predominant livelihood.

Existing studies also show that poverty reduction and economic development bring rapid consumption of resources and environmental damage^26^, and poverty-stricken counties overlap highly with ecologically fragile areas geographically and spatially^27,28^, which are more likely to cause serious environmental quality deterioration problems in the process of poverty alleviation. At the same time, the policy of poverty alleviation requires ecological poverty alleviation, so it is of great significance to study the impact of poverty alleviation policy on the environment in poor areas to achieve sustainable development. Dai et al.^29^ compared the poverty alleviation outcomes in two Chinese Provinces, Anhui and Guizhou provinces, and concluded that smaller, localized projects had a greater impact on reducing poverty than larger-scale infrastructure and industrial developments. Similarly, in their research, Fan and Chang-Kang^30^ argued that the Chinese government’s emphasis on building major intercity highways did not alleviate poverty as efficiently as investing in the development of simpler roads in isolated regions would have. Yang et al.^31^ utilized a meta-frontier undesirable dynamic two-stage DEA to assess anti-poverty policy efficiencies across Chinese provinces and underscored regional disparities in policy efficiency and highlighted the superior performance of poverty reduction efforts over agricultural productivity. While Yang et al.^31^ provided a comprehensive analysis of policy efficiencies across Chinese provinces, their study primarily focuses on the economic aspects of these policies, with less emphasis on the fiscal support through central government and institutions at the local level on sustainable agricultural infrastructure development and environmental determinants that could significantly impact agricultural productivity and, consequently, poverty reduction efforts.

Thus, we extend the meta-frontier undesirable dynamic two-stage DEA framework used by Yang et al.^31^ to include the index of infrastructure for sustainable agricultural development and environmental factors. In addition, very little research has paid attention to the impact of poverty alleviation policies on the number of rural residents guaranteed minimum subsistence allowance on the ground. This paper sought to bridge this gap by exploring how government efficiency in China shapes the distribution of fiscal support through central government and institutions at the local level on infrastructure for sustainable agricultural development and the number of rural residents guaranteed minimum subsistence allowance.

### Agricultural production efficiency

From 2012 to 2016, China’s agricultural development was in the implementation period of the 12th Five-Year Plan. At that time, the Chinese government mainly focused on improving agricultural productivity and farmers’ income, as well as strengthening rural infrastructure construction. These policies laid the foundation for agricultural development in the 14th Five-Year Plan of China. However, in the past decade, China’s agricultural development direction has undergone significant changes. From the basis of simply improving productivity and income in the 12th Five-Year Plan to the 14th Five-Year Plan, China’s agricultural development has shifted towards a greater emphasis on modernization, quality and efficiency, and competitiveness. This shift in focus highlights the need for a dynamic analysis of China’s agricultural production efficiency.

The discourse on agricultural production efficiency robustly positions government interventions as pivotal elements for enhancing sectoral efficiency^32,33^. These studies highlight the transformative potential of policy frameworks and technological advancements in boosting agricultural outputs and, consequently, fostering economic development within rural landscapes. Ambali^32^ explicates the direct correlation between policy induced modernization efforts and the increase in agricultural productivity in sub-Saharan Africa, while Apezteguía^33^ outlines the positive impacts of modernization initiatives on the agrarian economies in Argentina. Complementing these perspectives, Sikandar et al.^34^ expand the narrative by examining the panel data across developing economies in Latin America, Asia, and Eastern Europe, they underscores the critical influence of integrating developing economies into global food supply chains and highlights the necessity of capital infusion for achieving sustainable growth in the agricultural sector and effective poverty reduction.

In the context of China, various scholars contribute to this narrative by examining the specificities of government policies on agricultural efficiency and poverty reduction^35–37^. Jiang et al.^38^ analyze the role of rural collective economy policies in enhancing common prosperity, revealing how farmland transfer and scale operation under these policies significantly improve agricultural efficiency and rural prosperity. Chen et al.^35^ further refine this analysis by distinguishing the effects of farmland transfers on poverty vulnerability among smallholder households. They identify a significant reduction in poverty vulnerability households, underscoring the complexities of farmland transfer as a non-uniform poverty alleviation tool. Wang^39^ found that market restrictions and household characteristics significantly influence efficiency, with education, family size, and income playing a key role. Hu & McAleer^36^ estimated an increase in production efficiency over time but noted a growing gap between affluent coastal regions and the western hinterland. Complementing this narrative, Yan et al.^37^ delve into the “Poverty Alleviation through Agriculture Project,” highlighting the challenges of translating enhanced agricultural efficiency into effective poverty alleviation, thereby stressing the need for a more nuanced understanding of the dynamics between agricultural productivity and poverty reduction mechanisms.

Despite these insights, a discernible gap persists in the literature concerning the direct translation of efficiency gains into tangible poverty reduction outcomes, particularly amidst the exacerbating challenges posed by climate change^40^. Noack and Larsen^40^ critically assess the interplay between farm size, productivity, and poverty, underscoring the complex dynamics between these variables and the overarching influence of climatic variabilities. Their research suggests that while agricultural efficiency is a crucial component of rural economic development, its direct impact on poverty alleviation is mediated by a multitude of factors, including climate change, which remains under explored.

This gap highlights the need for a nuanced exploration of how agricultural efficiency gains, underpinned by government policies can translate into poverty reduction, particularly within the context of climate change. Our study aims to bridge this gap by integrating insights from both global and Chinese-specific literature, delving into the multifaceted relationship between agricultural efficiency, government interventions, and poverty outcomes within rural landscapes, especially under the looming shadow of climate-induced uncertainties.

### Impact of rainfall on agricultural production and government poverty reduction

As a meteorological factor in the natural environment, rainfall is highly random^41^^,^^42^. It’s well established in the literature that rainfall has a significant impact on the agricultural production system that we analyze, thus satisfying exogeneity and correlation.

The impact of rainfall on agricultural production and government poverty reduction is a complex issue. Hagos et al.^43^, Kyei-Mensah et al.^44^ and Fei and Lin^45^ highlight the importance of agricultural water management technologies in mitigating the negative effects of rainfall variability on poverty and food insecurity. Similarly, Huang et al.^46^ and Abdul Rahim^47^ both found that irrigation and soil and water conservation significantly contribute to increased yields and incomes, particularly in poor areas, and help in poverty mitigation. However, Asiimwe^48^ emphasize the need for a multi-faceted approach, including education, infrastructure, and land policies, to address the vulnerability of agricultural households to rainfall shocks and reduce food poverty. In the same vein, the research of Liu and Zeng^49^ emphasized the importance of the agricultural products circulation infrastructure and effective government policies in poverty reduction,however, like the research of Asiimwe^48^ overlooked the impact of rainfall. Cook et al.^50^ stated the problem of rainwater and agricultural production is more about the intensity and timing of the rainfall rather than the scarcity of rainfall in terms of the poverty-stricken Gangsu Province in China. Xue et al.^28^ showed that the spatial distribution of the agricultural water environmental efficiency in China is uneven, showing a gradual decrease from east to west. The results also showed that there is still a large gap in the research on the rainfall impact on agriculture and the economic situation of the whole country, considering China’s vast and diversified territory. While studies have shown significant impacts of rainfall variability on agricultural production^43,44^, there is a lack of research on integrating these impacts into government poverty alleviation policies and agricultural production strategies to enhance overall sustainability and efficiency^51^.

While the existing literature collectively highlights the nuanced relationship between rainfall, agricultural production, and government poverty reduction, it lacks a comprehensive perspective when discussing government poverty alleviation policies, agricultural production efficiency, and the impacts of climate change (especially rainfall) on these factors, particularly in terms of how these factors interact to affect sustainable agricultural development and poverty reduction. We thus add rainfall as an exogenous variable into the research model to explore the effects and fill the gap in the literature. Furthermore, as indicated by Xue et al.^28^, given the extensive and varied landscape of China, we categorize China’s 27 provinces into southern and northern regions according to the climatic boundary Qinling-Huaihe line for a wholistic comparative study of environmental, economic, and governmental efficiency.

### Meta DDF

Chung et al.^52^ introduced the concept of an output-orientated distance function (DDF), which is an extended directionally orientated output distance function. The traditional DDF, as a radial measurement model, usually overestimates efficiency values. To address this issue, Chen et al.^53^ established a non-oriented directional distance function, which leads to a more reasonable and accurate estimation of the efficiency value Fare et al.^54^ proposed the Network Data Envelopment Analysis (Network DEA) model. Compared with the traditional DEA model that treats production technologies as black boxes, the Network DEA model focuses on illustrating these production technologies and explores in detail the input allocations and intermediate outputs that may have a potential impact on the In contrast to traditional DEA models that treat production technologies as black boxes, network DEA models focus on describing these production technologies and exploring in detail the potential impact that input allocations and intermediate outputs may have on the production process, rather than treating them as black boxes that cannot be measured. A basic type of network structure is the parallel system, where the DMUs of the production process consist of sub-units Kao^55^ investigates the relationship between the underestimation of the efficiency values of the sub-systems and the underestimation of the efficiency of the whole system and proposes the use of a parallel DEA model to compute the overall and partial efficiency values Färe and Grosskopf^56^, using Dsingle- and two-level hierarchical models were proposed to calculate the efficiency of hierarchical and networked system subsystems, where each DMU was set up as a consecutive parallel subunit. In the network DEA model, a dynamic approach is allowed.

Therefore, extending the literatures reviewed, this study novelly include the index of infrastructure for sustainable agricultural development and environmental factors and adopt the DDF model as a research methodology. This allows more relevant, accurate, and reasonable evaluation results to make it more realistic which Fare and Grosskopf^57^ failed to take into account the persistent effects across periods with different stages of parallel systems while considering different production technologies. To correct this deficiency, this study revised the traditional DDF model and combined the parallel DEA model of Shannon^58^ Entropy and Kao^55^ and the concept of common frontier (Meta frontier) proposed by O’Donnell et al.^59^ to present the Meta entropy parallel two-stage dynamic DDF empirical study. The aim of this study is to evaluate the efficiency stage of agricultural production (SDG2, which aims to ensure food security) and the efficiency stage of government poverty alleviation (SDG1, which aims to eliminate all forms of poverty) in 27 provinces in China, in order to avoid underestimation or overestimation of efficiency values. Specifically, this paper first introduces the Entropy method and then builds a Meta-frontier entropy parallel two-stage dynamic DDF model.

## Research methodology

### The entropy method

#### Detailed indicators

In this study, on the basis of fully considering the impact of governmental inputs on agricultural infrastructure, and referring to the existing research results^60–62^, in the second stage of the model, the “index of infrastructure for sustainable development of agriculture” was used as the expected output to be included in the analytical framework. In the second stage of the model, the expected output of “Sustainable Agricultural Development Infrastructure Index” is incorporated into the analytical framework, which consists of five secondary indicators, including postal and telecommunication facilities, ecological and environmental facilities, water resources and water supply and drainage facilities, energy and power facilities, and road transportation facilities, and covers 22 specific sub-indicators. The subjective assignment method relies on the intention of the decision maker when assigning weights to the indicators, which is not objective, so the Shannon^58^ Entropy method was used to synthesize the line item indicators into a single value (Table 1 is detailed indicators).

**Table 1:** Sustainable agricultural development infrastructure index.

Normative layer	Indicator layer	Indicator stratum II	Unit
Level of infrastructure for sustainable agricultural development	Post and telecommunications infrastructure	telephone penetration	%
		Internet penetration	%
		Volume of telecommunication services	billions
		Density of post office distribution	(units/km2 )
	Ecological and environmental infrastructure	Non-hazardous domestic waste disposal rate Public toilets per 10,000 population Daily sewage treatment capacity Parkland per capita green coverage	% collars cubic metre metre %
	Water resources and drainage infrastructure	Density of water pipes Density of drainage pipes Total water supply Water penetration rate	Km/km2 Km/km2 cubic metre %
	Energy power infrastructure	Total gas supply	cubic metre
		electricity supply	billion kilowatt-hours
		Gas pipeline density Gas penetration rate	Km/km2 %
	Transport and logistics infrastructure	Road area per capita road network density	metre Km/km2
		Operational line network density Public transport vehicles per 10,000 population Total public transport passenger traffic	Km/km2 classifier for wheeled vehicles ten thousand visits

Source: Geng and Li^61^.

#### Entropy method steps

Step 1: Data standardization. Detailed indicators from the Government’s Poverty Reduction Phase output item “ Sustainable Agricultural Development Infrastructure Index “ for the 27 provinces of China were calculated using the following formula.

1 $$\begin{array}{*{20}c} {r_{mn} = \frac{{\mathop {\max }\limits_{m} x_{mn} - x_{mn} }}{{\mathop {\max }\limits_{m} x_{mn} - \mathop {\min }\limits_{m} x_{mn} }} \left( {m = 1, \ldots ,{ }27;{ }n = { }1,{ } \ldots ,N} \right)} \\ \end{array}$$

where $r_{mn}$ is the standardised value of the nth indicator for the mth province, and $\mathop {\min }\limits_{m} x_{mn}$ is the minimum value of the nth indicator for the mth province; $\mathop {\max }\limits_{m} x_{mn}$ is the maximum value of the nth indicator for the mth province.

Step 2: Sum up the standardised values for each sub-indicator.

2 $$\begin{array}{*{20}c} {P_{mn} = \frac{{R_{mn} }}{{\mathop \sum \nolimits_{m = 1}^{27} R_{mn} }} \left( {m = 1, \ldots ,{ }27;{ }n = { }1,{ } \ldots ,N} \right)} \\ \end{array}$$

where $P_{mn}$ denotes the proportion of the standardised value of the nth indicator to the sum of the standardised values for the m provinces.

Step 3: Calculate the entropy value for the nth indicator ($e_{n}$).

3 $$\begin{array}{*{20}c} {e_{n} = - \left( {\ln 27} \right)^{ - 1} \mathop \sum \limits_{m = 1}^{27} \left[ {P_{mn} \ln \left( {P_{mn} } \right)} \right] \left( {m = 1, \ldots ,{ }27;{ }n = { }1,{ } \ldots ,N} \right)} \\ \end{array}$$

Step 4: Calculate the weight of the nth index.

4 $$\begin{array}{*{20}c} {w_{n} = \frac{{1 - e_{n} }}{{\mathop \sum \nolimits_{n = 1}^{N} \left( {1 - e_{n} } \right)}} \left( {n = 1, \ldots ,N} \right)} \\ \end{array}$$

The Entropy method described above allows us to introduce a dynamic DDF model of the common boundary network under exogenous DEA. The details of the model are described below:

### Meta-frontier entropy network dynamic DDF under exogenous DEA model

Our assumption is that all provinces (P) consist of decision-making units (DMUs) due to differences in labor, capital, land, technology, or government governance efficacy, where cluster G contains P = P1 + P2 + ··· + Pg. We further set two phases of period t time (t = 1,…, T), respectively, and in each of them In each period, there are two different phases (i.e., efficiencies correspondingly): the efficiency of agricultural production and the efficiency of government support to agriculture.

There are I inputs in the agricultural production stage $x_{ij1}^{tm} ,{ }\left( {i = 1, \ldots ,{\text{I}}} \right)$ and produces D desired outputs $E1_{dj}^{tm} \left( {{\text{d}} = 1 \ldots \ldots {\text{D}}} \right)$ and produce D desired outputs; in the government expenditure phase there are C additional inputs $f_{cj}^{tm} \left( {c = 1, \ldots ,{\text{C}}} \right)$ in the government spending stage and produce V desired outputs $E2_{vj}^{tm} \left( {{\text{v}} = 1 \ldots \ldots {\text{V}}} \right)$ and G non-desired outputs $U_{gj}^{tm} \left( {g = 1 \ldots \ldots G} \right)$, where ${ }z_{ej}^{tm} \left( {e = 1 \ldots \ldots E} \right)$ are intermediate outputs that link (links) the agricultural production stage (Stage1) to the government farm support stage (Stage2).Finally ${ }L_{hj}^{tm} (h = 1 \ldots .H$ ) is a carry-over factor and has Q exogenous variables ${ }O_{qj}^{tm} \left( {q = 1 \ldots .Q} \right)$ . Under the common boundary, the decision unit z can choose the final output that is most favorable to its maximum value, therefore, the efficiency of DMUz under the common boundary can be solved by the following linear programming process.

Objective function:

DMU efficiency is:

5 $$\begin{array}{*{20}c} { \max {\text{Meta frontier efficiency}}\left( {{\text{MFE}}} \right) = \mathop \sum \limits_{g = 1}^{G} \mathop \sum \limits_{t = 1}^{T} {\upgamma }_{tg} \left( {w_{1g}^{t} \theta_{1g}^{t} + w_{2g}^{t} \theta_{2g}^{t} } \right)} \\ \end{array}$$

Subject to:

Production efficiency stage and Government Poverty Reduction stage

6 $$\begin{gathered} \mathop \sum \limits_{g = 1}^{G} \mathop \sum \limits_{j}^{n} \lambda_{jg}^{t} X_{ijg}^{t} \le \theta_{1g}^{t} X_{ijg}^{t} \forall i\forall t \mathop \sum \limits_{g = 1}^{G} \mathop \sum \limits_{j}^{n} \mu_{jg}^{t} Z_{djg}^{t} \le \theta_{2g}^{t} Z_{djg}^{t} \forall d\forall t \hfill \\ \mathop \sum \limits_{g = 1}^{G} \mathop \sum \limits_{j}^{n} \lambda_{jg}^{t} z_{djg}^{t} \le \theta_{1g}^{t} z_{djg}^{t} \forall d\forall t \mathop \sum \limits_{g = 1}^{G} \mathop \sum \limits_{j}^{n} \mu_{jg}^{t} y_{rjg}^{vt} \ge \theta_{2g}^{t} y_{rjg}^{vf} \forall r\forall t \hfill \\ \mathop \sum \limits_{g = 1}^{G} \mathop \sum \limits_{j}^{n} \lambda_{jg}^{t} q_{kjg}^{t} \ge \theta_{1g}^{t} q_{jg}^{t} \forall k\forall t \mathop \sum \limits_{g = 1}^{G} \mathop \sum \limits_{j}^{n} \mu_{jg}^{t} y_{rjg}^{bt} \le \theta_{2g}^{t} y_{rjg}^{bt} \forall r\forall t \hfill \\ \mathop \sum \limits_{g = 1}^{G} \mathop \sum \limits_{j}^{n} \lambda_{jg}^{k} \le 1 \forall t \mathop \sum \limits_{g = 1}^{G} \mathop \sum \limits_{j}^{n} \mu_{jg}^{t} w_{cjg}^{t} \le \theta_{2g}^{t} w_{cjg}^{t} \forall c\forall t \hfill \\ \lambda_{j}^{t} \ge 0\forall j \forall t \mathop \sum \limits_{g = 1}^{G} \mathop \sum \limits_{j}^{n} \mu_{jg}^{t} = 1 \forall t \hfill \\ \mu_{j}^{t} \ge 0 \forall j\forall t \hfill \\ \end{gathered}$$

Exogenous variables:

7 $$\begin{array}{*{20}c} {\mathop \sum \limits_{g = 1}^{G} \mathop \sum \limits_{j = 1}^{n} \lambda_{jg}^{t} Z_{djg}^{t} = \mathop \sum \limits_{g = 1}^{G} \mathop \sum \limits_{j = 1}^{n} \mu_{jg}^{t} Z_{djg}^{t} \forall d\forall t} \\ \end{array}$$

Link of the two stages.

8 $$\begin{array}{*{20}c} {\mathop \sum \limits_{g = 1}^{G} \mathop \sum \limits_{j = 1}^{n} \lambda_{jg}^{t - 1} c_{hjg}^{t} = \mathop \sum \limits_{g = 1}^{G} \mathop \sum \limits_{j = 1}^{n} \lambda_{jg}^{t} c_{hjg}^{t} \forall h\forall t} \\ \end{array}$$

Among them, the ${\upgamma }_{t}$ are the weights in period t, while $w_{1}^{t}$ and $w_{2}^{t}$ are the weights for the agricultural production efficiency stage and the government poverty reduction efficiency stage, respectively. Thus, for each time period $w_{1}^{t}$ , $w_{2}^{t}$ , ${\upgamma }_{t} \ge 1$ , and $\mathop \sum \nolimits_{g = 1}^{G} \mathop \sum \nolimits_{t = 1}^{T} {\upgamma }_{tg} = 1$ .

We can calculate the following three efficiency groups by linear programming.

(1) Stage efficiency.

The efficiency of stage L (L = 1, 2) of the DMU to be evaluated is appraised relative to each period t (t = 1,…, T). The stage efficiency can be expressed as:

Stage1: Agricultural productivity values

9 $$\begin{array}{*{20}c} {\rho_{1}^{{t^{*} }} = 1 - \theta_{l}^{{t^{*} }} ;l = 1,2;t = 1,2, \ldots ,T} \\ \end{array}$$

Stage 2: Government efficiency value for poverty reduction

10 $$\begin{array}{*{20}c} {\rho_{2}^{{t^{*} }} = 1 - \mathop \sum \limits_{t = 1}^{T} \gamma_{t} \theta_{l}^{{t^{*} }} ;l = 1,2} \\ \end{array}$$

(2) Period efficiency value.

In this group, the overall efficiency of each period t of the DMU being evaluated is.

11 $$\begin{array}{*{20}c} {\rho^{{t^{*} }} = w_{1}^{t} \rho_{1}^{{t^{*} }} + w_{2}^{t} \rho_{2}^{{t^{*} }} ;t = 1,2, \ldots ,T} \\ \end{array}$$

(3) Overall efficiency value.

The overall efficiency of the DMUs evaluated in this group. The overall efficiency is obtained by weighting the period efficiency values over t, e.g.

12 $$\begin{array}{*{20}c} {\rho^{*} = \mathop \sum \limits_{t = 1}^{T} \gamma_{t} \rho^{{t^{*} }} } \\ \end{array}$$

### Group-Frontier Efficiency (GFE)

Under the cluster boundary, the decision unit can also choose the final output that is most favourable to its maximum value, therefore, the efficiency of the DMU under the cluster boundary can be solved by the following linear programming process.

(a) Objective function

The DMU efficiency is

13 $$\begin{array}{*{20}c} {max{\text{ Group}}\;{\text{frontier}}\;{\text{efficiency}}\;{\text{(GFE) }} = \mathop \sum \limits_{t = 1}^{T} \gamma_{t} \left( {w_{1}^{t} \theta_{1}^{t} + w_{2}^{t} \theta_{2}^{t} } \right)} \\ \end{array}$$

Subject to

Production efficiency stage and Government Poverty Reduction stage

$$\mathop \sum \limits_{j}^{n} \lambda_{j}^{t} X_{ij}^{t} \le \theta_{1}^{t} X_{ij}^{t} { }\forall i\forall t{ }\mathop \sum \limits_{j}^{n} \mu_{j}^{t} Z_{dj}^{t} \le \theta_{2}^{t} Z_{dj}^{t} { }\forall d\forall t$$

$$\mathop \sum \limits_{j}^{n} \lambda_{j}^{t} z_{dj}^{t} \le \theta_{1}^{t} z_{dj}^{t} { }\forall d\forall t{ }\mathop \sum \limits_{j}^{n} \mu_{j}^{t} y_{rj}^{vt} \ge \theta_{2}^{t} y_{rj}^{vt} { }\forall r\forall t$$

$$\mathop \sum \limits_{j}^{n} \lambda_{j}^{t} q_{kj}^{t} \ge \theta_{1}^{t} q_{kj}^{t} { }\forall k\forall t{ }\mathop \sum \limits_{j}^{n} \mu_{j}^{t} y_{rj}^{bt} \le \theta_{2}^{t} y_{rj}^{bt} { }\forall r\forall t$$

$$\mathop \sum \limits_{j}^{n} \lambda_{j}^{k} \le 1{ }\forall t{ }\mathop \sum \limits_{j}^{n} \mu_{j}^{t} w_{cj}^{t} \le \theta_{2}^{t} w_{cj}^{t} { }\forall c\forall t$$

$$\lambda_{j}^{t} \ge 0{ }\forall j\forall t{ }\mathop \sum \limits_{j}^{n} \mu_{j}^{t} = 1{ }\forall t$$

14 $$\begin{array}{*{20}c} {\mu_{j}^{t} \ge 0 \forall j\forall t} \\ \end{array}$$

Exogenous variables.

15 $$\begin{array}{*{20}c} {\mathop \sum \limits_{j = 1}^{T} \lambda_{1}^{t} b_{Uj}^{t} = \theta_{1}^{t} b_{U}^{t} \forall U\forall t} \\ \end{array}$$

Link of the two stages.

16 $$\begin{array}{*{20}c} {\mathop \sum \limits_{j = 1}^{n} \lambda_{j}^{t} Z_{dj}^{t} = \mathop \sum \limits_{j = 1}^{n} \mu_{j}^{t} Z_{dj}^{t} \forall d\forall t} \\ \end{array}$$

Link of the two periods:

17 $$\begin{array}{*{20}c} {\mathop \sum \limits_{j = 1}^{n} \lambda_{j}^{t - 1} c_{hj}^{t} = \mathop \sum \limits_{j = 1}^{n} \lambda_{j}^{t} c_{hj}^{t} \forall h\forall t} \\ \end{array}$$

Among them, the ${\upgamma }_{t}$ are the weights in period t, while $w_{1}^{t}$ and $w_{2}^{t}$ are the weights for the agricultural production efficiency stage and the government poverty reduction efficiency stage, respectively. Thus, for each time period $w_{1}^{t}$ , $w_{2}^{t}$ , ${\upgamma }_{t} \ge 1$ , and $\mathop \sum \nolimits_{g = 1}^{G} \mathop \sum \nolimits_{t = 1}^{T} {\upgamma }_{tg} = 1$ .

We calculated the following three efficiency groups by linear programming.

(1) Stage efficiency

The efficiency of stage L (L = 1, 2) of the DMU to be evaluated is appraised relative to each period t (t = 1, . . . , T). The stage efficiency can be expressed as:

Stage1: Agricultural Productivity values

18 $$\begin{array}{*{20}c} {\rho_{1}^{{{\text{tg}}}} = 1 - \theta_{l}^{{{\text{tg}}^{*} }} ;l = 1,2;t = 1,2, \ldots ,T} \\ \end{array}$$

Stage2: Government efficiency value for poverty reduction

19 $$\begin{array}{*{20}c} {\rho_{2}^{tg} = 1 - \mathop \sum \limits_{t = 1}^{T} \gamma_{t} \theta_{t}^{{tg^{*} }} ;l = 1,2} \\ \end{array}$$

(2) Period efficiency value.

In this group, the overall efficiency of each period t of the DMU being evaluated is.

20 $$\begin{array}{*{20}c} {\rho^{tg} = w_{1}^{t} \rho_{1}^{tg} + w_{2}^{t} \rho_{2}^{tg} ;t = 1,2, \cdots ,T } \\ \end{array}$$

(3) Overall efficiency

The overall efficiency of the DMUs evaluated in this group. The overall efficiency is obtained by weighting the period efficiency values over t, e.g.…

21 $$\begin{array}{*{20}c} {\rho^{{{*}g}} = \mathop \sum \limits_{t = 1}^{T} \gamma_{t} \rho^{tg} } \\ \end{array}$$

### Technology gap ratio (TGR)

The technical efficiency of Meta-frontier (MFE) is less than the technical efficiency of group-frontier (GFE) since g groups are included in the Meta-frontier model. The ratio is the Technology Gap Ratio (TGR):

22 $$\begin{array}{*{20}c} {TGR = \frac{{\rho^{*} }}{{\rho^{{{*}g}} }} = \frac{MFE}{{GFE}}} \\ \end{array}$$

### Inputs, unexpected outputs, and expected output efficiencies

We adopted the total factor energy efficiency indicators proposed by Hu and Wang to overcome the possible bias of traditional efficiency indicators. These include eight key efficiency indicators, namely, the sown area of crops, the number of legal entities in agriculture, and financial expenditure as inputs, the gross agricultural product, the disposable income per capita of rural residents, the level of infrastructure for sustainable agricultural development, and the number of people covered by the minimum subsistence guarantee for rural residents as outputs, and the number of large and medium-sized tractors used in agriculture as a carry-over variable is also taken into account. Here, the symbol “i” stands for area, and “t” stands for time. The formula for calculating the efficiency value of the key indicators for each DMUit is as follows:

23 $$\begin{array}{*{20}c} {{\text{Input}}\;{\text{efficiency }} = \frac{{{\text{Target}}\;{\text{input}}}}{{{\text{Actual}}\;{\text{input}}}}} \\ \end{array}$$

24 $$\begin{array}{*{20}c} {{\text{Unexpected}}\;{\text{output}}\;{\text{efficiency }} = \frac{{{\text{Target}}\;{\text{Unexpected}}\;{\text{output}}}}{{{\text{Actual}}\;{\text{Unexpected}}\;{\text{output}}}}} \\ \end{array}$$

25 $$\begin{array}{*{20}c} {{\text{Expected}}\;{\text{output}}\;{\text{efficiency }} = \frac{{{\text{Actual}}\;{\text{Expected}}\;{\text{output}}}}{{{\text{ Target}}\;{\text{Expected}}\;{\text{output}}}}} \\ \end{array}$$

If the targeted inputs are equal to the actual inputs, the efficiency is 1; however, if the targeted inputs are less than the actual inputs, the efficiency is less than 1, indicating an overall lower efficiency. If the targeted expected output is equal to the actual expected output, the efficiency is 1; however, if the targeted expected output is greater than the actual expected output, the efficiency is less than 1, indicating overall inefficiency. If the targeted non-desired output is equal to the actual non-desired output, the efficiency is 1; however, if the targeted unexpected output is less than the actual unexpected output, the efficiency is less than 1, indicating overall inefficiency.

## Empirical analysis

### Data and variables

Based on data availability and the results of the final model, we collected data for 2016–2020. Since the central financial poverty alleviation funds do not cover Beijing, Tianjin, and Shanghai, and the data for Tibet are seriously missing, we finally chose to use panel data to conduct an empirical study on 27 provinces, autonomous regions, and municipalities (excluding Hong Kong, Macao, and Taiwan) in China, with the raw data from the China Statistical Yearbook, the China Agricultural Yearbook, and the China Rural Statistics Yearbook in previous years. Yearbook, China Agricultural Yearbook, and China Rural Statistics Yearbook. The exogenous variable rainfall was obtained from the China National Meteorological Information Database. Details of the variables used in the study are shown in Table 2:

**Table 2:** Input and output variables.

Stage	Variable Unit		
Stage 1	Input	Crop sown area	thousand hectares
	Input	Agricultural business entity	10,000
	Output	Agricultural GDP	100 million RMB
	Output	Per capita disposable income of rural residents	10,000 RMB
	Link	Per capita disposable income of rural residents	10,000 RMB
Stage 2	Input	Financial support for agriculture	100 million RMB
	Output	Sustainable Agricultural Development Infrastructure Index	–
	Output	Number of rural residents guaranteed minimum subsistence allowance	10,000 person
	Carryover	Number of agricultural large and medium-sized tractors	10,000
	Exogenous	Annual rainfall	100 million cubic meter

Data source: China Statistical Yearbook database, rainfall data from the National Meteorological Information Database.

Stage I: Agricultural Production stage

Input variables:

(A) Crop sown area (CA) is the area actually sown or transplanted with crops

(B) Agricultural business entity (ABE) is any individual or organization directly or indirectly engaged in the production, processing, marketing, and service of agricultural products. service of agricultural products.

Output variables:

(C) Agricultural GDP (AGDP) is the total amount of all products of agriculture, forestry, animal husbandry, and fishery expressed in monetary terms within a certain period (usually one year). It reflects the total scale and results of agricultural production. It reflects the total scale and results of agricultural production.

(D) Per capita disposable income of rural residents (RRDI) is the combination of final consumption expenditure and savings available to rural survey households, i.e., the income that survey households can use for discretionary purposes. Per capita disposable income of rural residents (RRDI) is the combination of final consumption expenditure and savings available to rural survey households, i.e., the income that survey households can use for discretionary purposes. Disposable income includes both cash and in-kind income.

Stage II: Government Poverty Reduction Stage

(E) Financial support for agriculture (FSA) is China’s national financial support for agriculture, rural areas, and farmers, the main means of national financial support for agriculture, rural areas, and farmers, and one of the important elements of the distribution relationship between the State and farmers. Financial support for agriculture (FSA) is China’s national financial support for agriculture, rural areas, and farmers, the main means of national financial support for agriculture, rural areas and farmers, and one of the important elements of the distribution relationship between the State and agriculture (FSA) is China’s national financial support for agriculture, rural areas and farmers, the main means of national financial support for agriculture, rural areas and farmers, and one of the important elements of the distribution relationship between the State and farmers, whose main forms of expression are capital input preferential policies and institutional construction.

Financial support for agriculture = central fiscal special funds for poverty alleviation + local government fiscal expenditure for agriculture.

(F) Sustainable Agricultural Development Infrastructure Index (ISA) is a comprehensive evaluation indicator synthesized by the entropy method: (1) postal and telecommunication facilities; (2) ecological facilities; (3) water resources, water supply, and drainage facilities; (4) energy and power facilities. (1) postal and telecommunication facilities; (2) ecological facilities; (3) water resources, water supply and drainage facilities; (4) energy and power facilities; and (5) road transportation facilities. (3) water resources, water supply, and drainage facilities; (4) energy and power facilities; and (5) road transportation facilities.

(G) Number of rural residents guaranteed minimum subsistence allowance (MSA) is a livelihood protection system introduced by the Chinese Government for rural residents whose annual per capita net household income is below the local minimum subsistence standard. A straightforward explanation is that “low security” is the same as the minimum subsistence guarantee. A straightforward explanation is that “low security” is the same as the minimum subsistence guarantee.

Carryover

(H) Number of agricultural large and medium-sized tractors (ALMT) is the number of medium and large tractors used for agricultural production and other related farming activities.

Exogenous

(I) Annual rainfall(R) is a measure of how much precipitation falls on an area. Specifically, it is the depth to which liquid and solid (melted) precipitation falling from the sky to the ground has accumulated on the horizontal plane without evaporation, infiltration, or loss. Specifically, it is the depth to which liquid and solid (melted) precipitation falling from the sky to the ground has accumulated on the horizontal plane without evaporation, infiltration, or loss.

**Figure 1:**
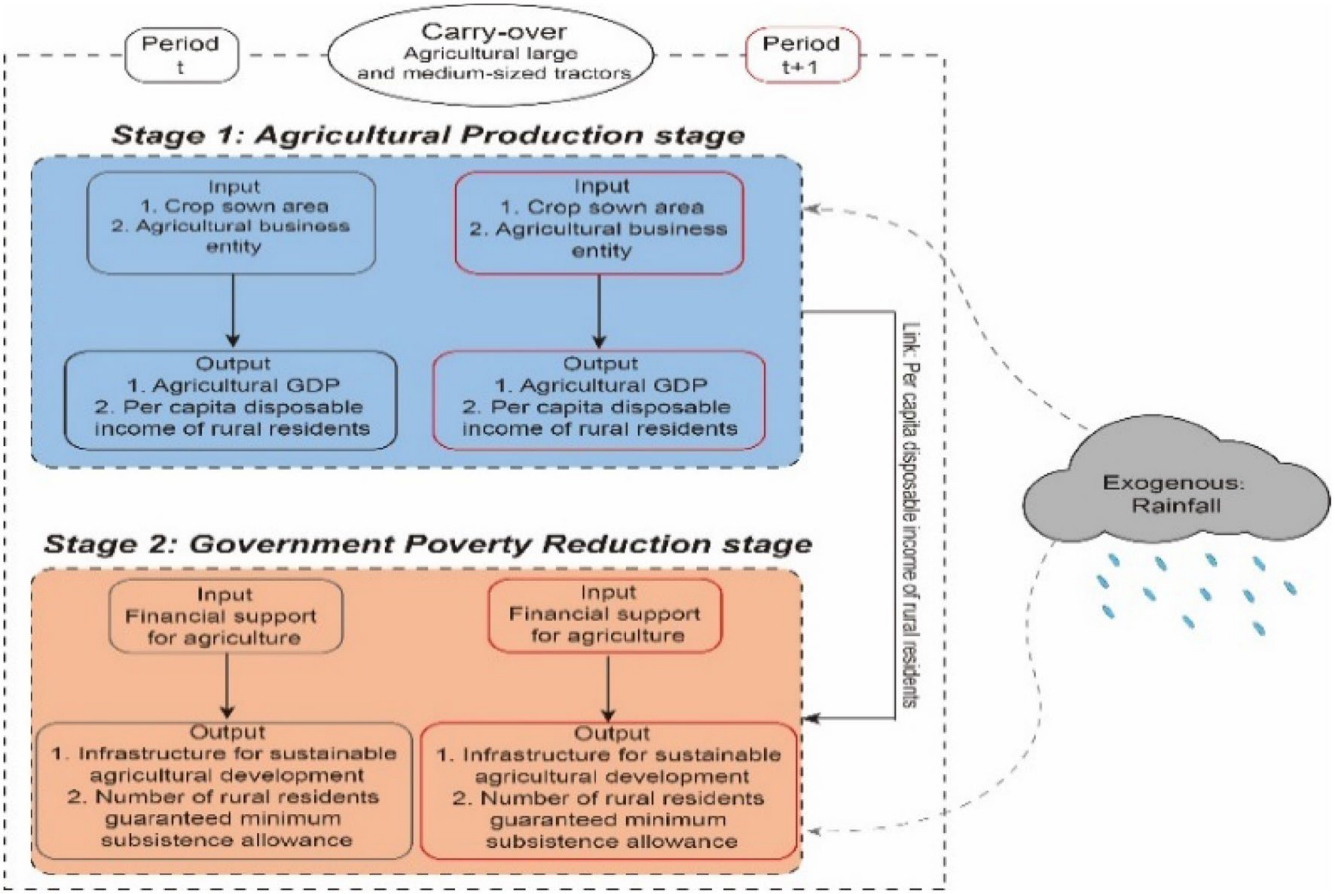
Model framework.

We used a modified non-expectation two-stage dynamic DDF model to analyze the estimation bias in the two-stage analysis using annual rainfall as an exogenous variable and disposable income per capita of rural residents as an intermediate linking variable. Based on these assumptions, we designed a Meta-frontier two-stage non-expectation dynamic DDF model under consideration of the effects of exogenous variables (see Fig. 1).

### Descriptive statistics of relevant indicators such as inputs and outputs

**Figure 2:**
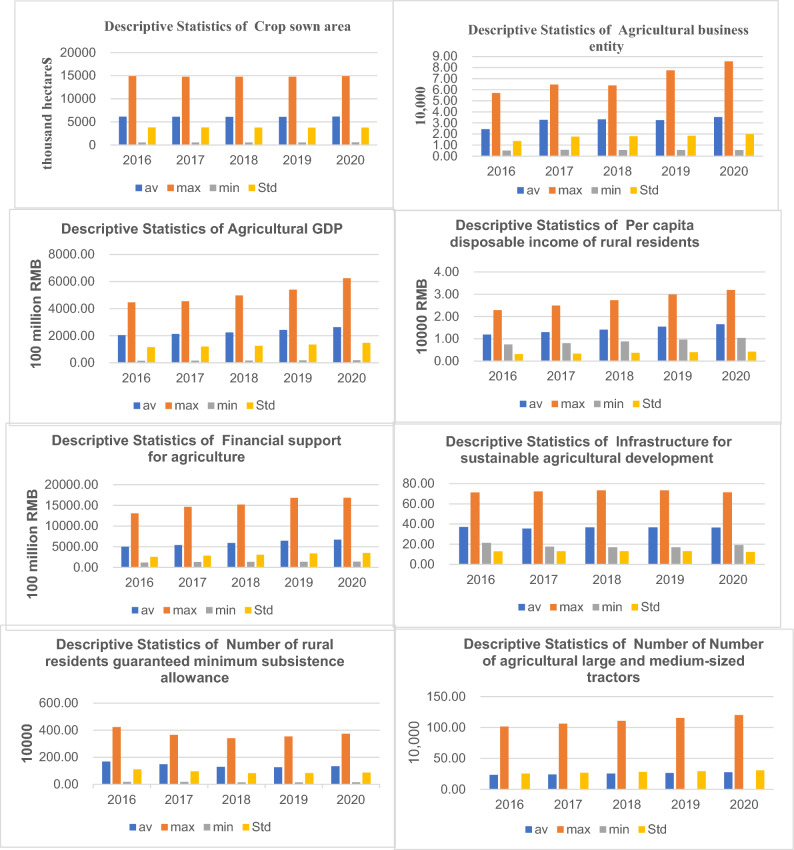
Input–output statistics (2016–2020).

Figure 2 presents input and output indicators, including input indicators for the agricultural production stage: total sown area of crops, number of legal entities in agriculture, and output indicators: gross agricultural product and disposable income per rural resident. The results of the statistical analysis of the Government’s additional input indicators for the poverty reduction stage: fiscal expenditure and output indicators: infrastructure for sustainable agricultural development and the number of rural inhabitants covered by the minimum subsistence guarantee.

The main reason for the slow growth of the average total sown area (inputs) of crops in 2016–2020 is affected by the 1.8 billion mu of arable land red line policy. In 2016, China’s Ministry of Land and Resources issued the Adjustment Programme for the Outline of the National Overall Land Use Plan (2006–2020), in which it adjusted the indicators of arable land retention, basic farmland protection area, and the total scale of land used for construction at the national level and in all provinces (autonomous regions and municipalities), requiring that by 2020 the national arable land retention will be more than 1865 million mu and The basic farmland protection area will be over 1.546 billion mu, and the total scale of construction land will be controlled within 40.71939 million hectares (610.79 million mu). From the perspective of Agricultural business entity (input), the average and maximum values have increased year by year, while the minimum value has not changed significantly in the rest of the years, except for a slight increase in 2017. The maximum and average values of Agricultural GDP have shown a trend of faster growth, while the minimum value has grown slowly and fluctuated slightly. Per capita disposable income of rural residents’ maximum value, average value, and minimum value all show an increasing trend year by year. Financial support for agriculture’s maximum value, average value, and minimum value continue to increase, and the gap between the maximum value and the minimum value is getting bigger and bigger, which indicates that Financial support for agriculture has maintained a certain degree of growth trend. In terms of Infrastructure for sustainable agricultural development, the maximum value fluctuates from 2019–2020, the index started to increase in 2016, but in 2020 the index fell and hit a new low. Both its minimum and average values also fluctuate slightly. The maximum value of the non-expected output Number of rural residents guaranteed minimum subsistence allowance shows a gradual decline in 2016–2018, but then rebounds in 2019–2020, and the performance of the minimum value also fluctuates slightly, but in terms of the overall performance of the average value 2016–2019 declined yearly but regressed to the 2018 level in 2020 due to the new crown epidemic. The maximum, minimum, and average values of the carry-over variable Number of agricultural large and medium-sized tractors all show a year-on-year upward trend and peak in 2020, which also reflects the increase in the degree of mechanization of agricultural production in China.

This paper takes the Qinling-Huaihe line as the regional demarcation line between the north and the south of China, and according to the latitude of the provinces and the difference in the degree of regional economic development, the 27 provinces of China are explored based on retaining the complete provincial administrative units, and the statistics of input–output indexes of each province are shown in Table 3:

**Table 3:** Division of regions in China.

Region	Provinces, municipalities, and autonomous regions
South	Fujian, Guangdong, Guangxi, Guizhou, Hainan, Hunan, Jiangsu, Jiangxi, Sichuan, Yunnan, Zhejiang, Chongqing
North	Anhui, Gansu, Hebei, Henan, Heilongjiang, Hubei, Jilin, Liaoning, Inner Mongolia, Ningxia, Qinghai, Shandong, Shanxi, Shaanxi, Xinjiang

Table 4 shows a comparison of key input–output indicators for the two regions for 2016–2020. We see that the Northern Area’s average Agricultural business entity inputs increase year on year, while Southern Areas fluctuate slightly. In terms of average Crop sown area inputs, Northern Areas has a clear comparative advantage over Southern Areas. In stage 1, Southern Areas outperform Northern Areas in terms of average agricultural GDP and per capita disposable income of rural residents, while in stage 2, the additional financial support for agriculture inputs, Northern Areas are significantly more favorable than Northern Areas. Support for agriculture inputs, Northern Areas is larger than Southern Areas, while in terms of output performance Southern Areas’ desired output Infrastructure for sustainable agricultural development is higher than that of Northern Areas in 2016, except that it is higher than that of Northern Areas in 2016. Development lags behind Northern Areas in 2017–2020, except for a slight lead in 2016, while in terms of non-desired outputs Rural residents guaranteed minimum subsistence allowance, Southern Areas’ farm household performance of the exogenous variable Rainfall is significantly higher in Southern Areas than in Northern Areas.

**Table 4:** Input and output variables from 2016–2020 between Northern Areas and Southern Areas.

Year	Region	ABE	CA	AGDP	RRDI	FSA	ALMT	ISA	MSA	Rainfall
2016	South	2.70	5103.67	2044.92	1.18	4621.46	30.92	37.56	128.56	2905.57
	North	2.22	6969.26	2039.67	1.21	5275.23	17.27	36.75	199.99	1744.68
2017	South	3.51	5109.91	2121.45	1.27	4980.85	32.11	34.54	106.10	2822.71
	North	3.09	6927.57	2137.63	1.31	5786.14	17.93	36.43	182.15	1771.89
2018	South	3.42	5086.63	2223.64	1.38	5397.13	33.63	35.45	92.70	2739.84
	North	3.24	6918.47	2252.90	1.43	6330.09	18.86	37.76	158.40	1799.10
2019	South	3.25	5097.29	2355.37	1.51	5793.29	35.07	35.45	88.93	2656.98
	North	3.26	6915.43	2483.73	1.57	6977.25	19.73	37.76	157.44	1826.30
2020	South	3.40	5174.34	2572.21	1.61	6138.96	36.51	36.39	94.29	2574.17
	North	3.63	6956.69	2686.89	1.69	7160.81	20.60	36.70	164.17	1853.53

### Comparative analysis of the overall efficiency of 27 provinces under two scenarios

We evaluate each DMU while considering (R) and excluding (R*) the effect of rainfall. Without considering exogenous variables, there are seven provinces with an overall efficiency of 0.8 or higher, with three of them in the position of the south and four in the north. After considering the exogenous variable rainfall, there are nine provinces with an overall efficiency of at least 0.8, four of which are located in the South and five in the North. The problem of underestimation of efficiency values is improved when we discuss rainfall as an exogenous variable in the model. A comparative comparison between the southern and northern regions clearly shows that the efficiency is significantly improved when exogenous variables are considered. This is shown in Table 5:

**Table 5:** Efficiency of each province in the two scenarios, 2016–2020.

Cluster	DMU	Overall		2016		2017		2018		2019		2020	
		R	R*	R	R*	R	R*	R	R*	R	R*	R	R*
South	Fujian	0.852	0.728	0.901	0.901	0.911	0.592	0.931	0.931	0.941	0.645	0.578	0.569
	Guangdong	1.000	1.000	1.000	1.000	1.000	1.000	1.000	1.000	1.000	1.000	1.000	1.000
	Guangxi	0.730	0.730	0.580	0.580	0.571	0.571	0.499	0.499	1.000	1.000	1.000	1.000
	Guizhou	0.615	0.615	0.586	0.586	0.588	0.588	0.608	0.608	0.624	0.624	0.671	0.671
	Hainan	0.920	0.920	1.000	1.000	1.000	1.000	1.000	1.000	0.866	0.866	0.734	0.734
	Hunan	0.529	0.523	0.700	0.697	0.462	0.453	0.497	0.488	0.517	0.512	0.470	0.464
	Jiangsu	0.959	0.828	0.960	0.868	0.979	0.691	0.956	0.735	0.959	0.959	0.943	0.885
	Jiangxi	0.550	0.532	0.582	0.558	0.573	0.564	0.563	0.531	0.553	0.539	0.478	0.469
	Sichuan	0.541	0.533	0.627	0.623	0.499	0.488	0.491	0.490	0.538	0.534	0.549	0.528
	Yunnan	0.623	0.623	0.694	0.694	0.574	0.574	0.553	0.553	0.699	0.699	0.594	0.594
	Zhejiang	0.612	0.605	0.677	0.662	0.633	0.616	0.575	0.575	0.601	0.598	0.574	0.573
	Chongqing	0.442	0.420	0.451	0.451	0.480	0.480	0.429	0.429	0.449	0.388	0.399	0.351
	Mean	0.698	0.671	0.730	0.718	0.689	0.635	0.675	0.653	0.729	0.697	0.666	0.653
North	Anhui	0.460	0.445	0.531	0.498	0.432	0.432	0.437	0.423	0.469	0.449	0.430	0.424
	Gansu	0.869	0.869	1.000	1.000	1.000	1.000	1.000	1.000	0.699	0.699	0.644	0.644
	Hebei	0.577	0.420	0.681	0.500	0.528	0.421	0.573	0.391	0.574	0.408	0.530	0.379
	Henan	0.741	0.431	0.907	0.573	0.782	0.440	0.647	0.386	0.633	0.374	0.734	0.383
	Heilongjiang	0.656	0.623	0.950	0.854	0.644	0.597	0.614	0.595	0.578	0.576	0.494	0.494
	Hubei	0.510	0.470	0.531	0.480	0.446	0.435	0.511	0.470	0.569	0.516	0.494	0.447
	Jilin	0.614	0.607	0.770	0.770	0.646	0.617	0.565	0.563	0.565	0.561	0.522	0.522
	Liaoning	0.680	0.589	0.788	0.732	0.773	0.649	0.665	0.573	0.624	0.545	0.552	0.446
	Inner Mongolia	0.606	0.565	0.631	0.609	0.580	0.560	0.609	0.547	0.626	0.569	0.582	0.538
	Ningxia	1.000	1.000	1.000	1.000	1.000	1.000	1.000	1.000	1.000	1.000	1.000	1.000
	Qinghai	0.990	0.942	1.000	0.965	1.000	1.000	1.000	0.968	1.000	0.939	0.951	0.839
	Shandong	0.930	0.414	0.945	0.494	0.941	0.424	0.917	0.406	0.922	0.411	0.925	0.334
	Shanxi	0.520	0.520	0.571	0.571	0.606	0.606	0.520	0.520	0.471	0.471	0.433	0.433
	Shaanxi	0.563	0.482	0.687	0.582	0.528	0.454	0.558	0.462	0.568	0.472	0.473	0.442
	Xinjiang	0.995	0.942	0.994	0.994	0.983	0.983	1.000	1.000	1.000	0.953	1.000	0.781
	Mean	0.714	0.621	0.799	0.708	0.726	0.641	0.708	0.620	0.687	0.596	0.651	0.540

**Figure 3:**
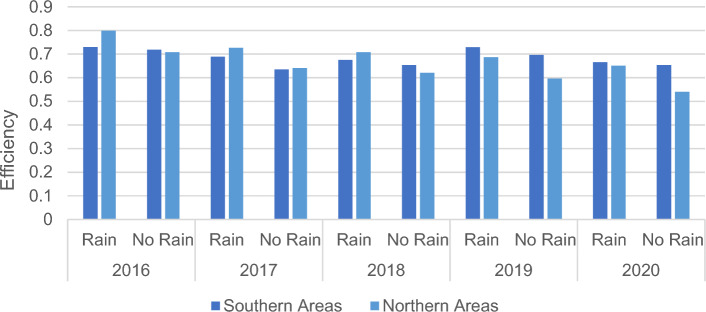
Meta-frontier Efficiency from 2016 to 2020 between the Northern Areas and Southern Areas. Rain: Rainfall.

As can be seen in Fig. 3, there is a mechanism of interaction between rainfall and the efficiency of sustainable agricultural development. Changes in rainfall can indirectly reflect changes in the climate environment of the region. In terms of spatial distribution China’s rainfall as a whole shows a wet and rainy southeast, gradually decreasing towards the inland northwest, and the vast inland northwestern region (except for individual areas of northwestern Xinjiang) is characterized by a dry climate with little precipitation. This results in an overall increase in efficiency in both the southern and northern regions after considering rainfall as an exogenous influence. After considering rainfall as an exogenous variable, the average total efficiency score of the northern region is higher than that of the southern region in 2016, 2017, and 2018, while in 2019 and 2020 it is the southern region that has a higher total efficiency score than the northern region. Both the Southern and Northern regions showed some fluctuations in their average total efficiency scores, implying that there is still more room for improvement in both places. Without considering rainfall as an exogenous variable, the average total efficiency score of the southern region dropped significantly from 0.72 in 2016 to 0.63 in 2017, recovered slightly in 2018 and 2019, and then dropped again in 2020 to remain the same as in 2018. In contrast, the average total efficiency score for the northern region shows a decreasing trend from year to year. It declined from 0.71 in 2016 to 0.54 in 2020.

### Efficiency analysis between the agricultural production stage and government poverty reduction stage

Southern and northern provinces are generally more efficient at the agricultural production stage than at the government poverty reduction stage. The overall efficiency value of the southern provinces in the agricultural production stage is 0.743, with room for improvement. In the following, we will analyze the efficiency performance of these two stages in terms of efficiency. Agricultural production efficiency is an important criterion for evaluating the efficiency of sustainable agricultural development, so it is necessary to analyze the efficiency of the agricultural production stage. Table 6 shows that the performance of the south and the north during the period of 2016–2020 is good, with an overall average value of 0.787, which is closely related to China’s “Three Rural” policy regulation, the improvement of agricultural mechanization and the rapid development of rural revitalization. The efficiency of the agricultural production stage in the northern provinces is significantly higher than that in the southern provinces, with an average efficiency of 0.822. This is mainly since the northern provinces are China’s main grain-producing areas, for example, according to the data published by the National Bureau of Statistics of China: in 2021, the total population of the seven grain-producing provinces in the north of China was 398 million people, which accounted for 28 percent of the country’s total population. The total grain output, however, is as high as 683.118 billion jin, accounting for about 50 percent of the country. In terms of topography, China’s major plains are concentrated in the northern region, while the terrain in the southern region is mostly hilly. In addition, the area of arable land, per capita area of arable land, the degree of agricultural mechanization, agricultural population, and per capita food ownership have all contributed to the increase in agricultural GDP, which has led to a better performance in terms of efficiency in the agricultural production phase.

**Table 6:** Efficiency of the Agricultural production stage between the Northern Areas and Southern Areas, 2016–2020.

Cluster	Mean	2016	2017	2018	2019	2020
South	0.743	0.791	0.714	0.692	0.775	0.742
North	0.822	0.898	0.791	0.828	0.816	0.778

Effective allocation of government financial resources and macroeconomic growth affect sustainable agricultural development, and numerous literatures have found that government intervention is an important factor contributing to the variability of regional economic development in China. We introduce indicators related to sustainable agricultural development (fiscal expenditure, Infrastructure for sustainable agricultural development, and the number of rural residents with minimum subsistence guarantee) into the input and output elements of the government’s poverty reduction stage to assess the Chinese government’s public service function and sustainability. As can be seen from Table 7, the overall average efficiency of the government’s poverty reduction stage is 0.666 lower than that of the agricultural production stage. From the evaluation results, the government poverty reduction efficiency of 0.679 in the southern provinces has a large room for improvement, and the efficiency value shows a U-shaped curve trend of decreasing and then increasing. The performance of the government’s poverty reduction efficiency in the northern provinces is 0.656, and its actual performance is also lower than expected compared to the more financial funds received from the Chinese government for poverty reduction, which actually contributes to the widening of the economic gap between the north and the south that has long been present in the Chinese economy.

**Table 7:** Efficiency of the Government poverty reduction stage between the Northern Areas and Southern Areas, 2016–2020.

Cluster	Mean	2016	2017	2018	2019	2020
South	0.679	0.697	0.675	0.673	0.632	0.718
North	0.656	0.709	0.687	0.652	0.632	0.599

An important feature of local government intervention in the microeconomic sector at all levels in China is administrative intervention in or control of the allocation and pricing of key factor markets within their jurisdictions, leading to distortions in factor markets as factor market reforms lag behind product market reforms. Specifically, due to the greater integration of southern China into the global industrial chain, supply chain, and value chain division of labor and trade system, under the dual effect of the historical tradition of being “relatively far away from the political center” and the mechanism of “external openness to force the reform of the internal market”, the factor market distortion is caused by the lagging behind of factor market reform compared with product market reform. The two factors are mutually reinforcing. In the Southern Plate, the government’s intervention and control of financial subsidies, financial markets, land markets, and other key factor resources are more in line with the principle of fair competition in the market, so that the development and operation mechanism of key factor markets are relatively perfect, and the dominant role of the market competition mechanism in the operation of the national economy is more prominent. On the contrary, in the face of the competitive pressure from the economic development and industrial development advantages of the southern sector, the government of the northern sector of China, in the process of attracting investments and promoting industrial development, is more inclined to adopt preferential policies and government subsidies that are contrary to the market competition mechanism, such as intervening in and controlling the distribution and pricing of specific key factors in the region. As a result, the market-oriented reform process, including product market-oriented reform and factor market-oriented reform, has lagged behind that of the Southern China region, which has resulted in the Southern China government’s poverty reduction efficiency being significantly higher than that of the Northern China government.

**Figure 4:**
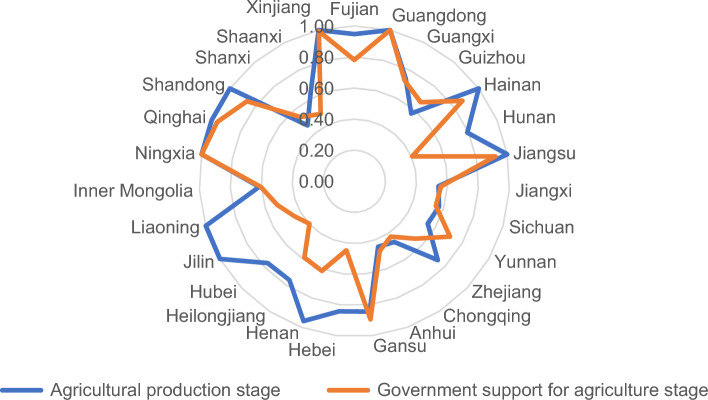
Efficiency of the Two-stage comparison of 27 provinces under exogenous variables rainfall.

As can be seen in Fig. 4, 13 of the 27 provinces are less efficient in the second stage than in the first stage, and 14 provinces are more efficient in the second stage than or the same as in the first stage. In the second stage, Yunnan province has the largest improvement in efficiency value of 0.712, which is 0.167 higher than the first stage. Jilin province has the largest regression in efficiency from an efficiency value of 1.000 in the agricultural production stage to 0.448 in the second stage. for both stages, Guangdong and Ningxia have an efficiency value of 1.

### Comparative analysis of the TGR in the region

**Figure 5:**
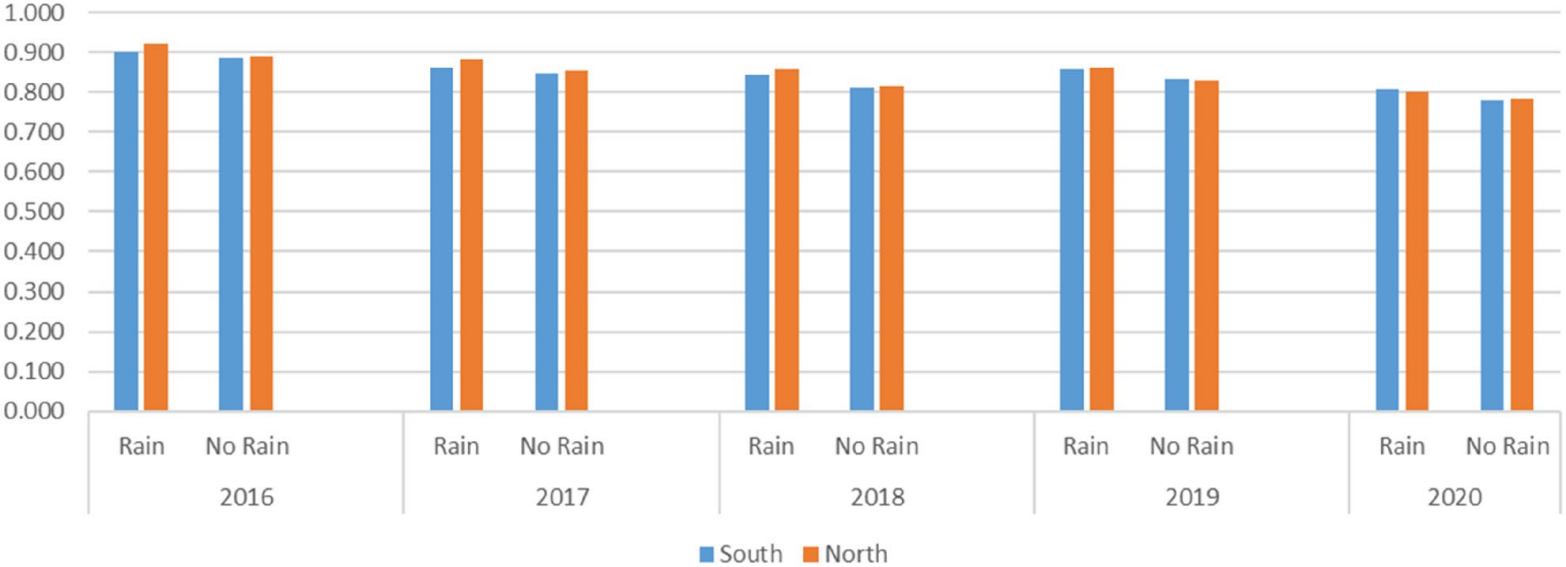
Technology gap from 2016 to 2020 between the North- and South Region.

As can be seen in Fig. 5, there is a gap between the technical variance rates of the northern region and the southern region when rainfall is considered as an exogenous variable. However, the gap is not very large, and the technical discrepancy rate in the northern region is above 0.8. In 2019, the northern region is higher than the southern region, but the gap is the smallest. On the whole, the rate of technological difference is higher in the northern regions of China, and there is more room for improvement. Therefore, the authorities should take measures to strengthen the sustainable governance of agricultural economic development and implement more intensive governance in the northern region.

### Analysis of the efficiency values of major inputs and outputs

As can be seen from Table 8, the efficiency performance of the main input–output indicators for 2016–2020 is inconsistent. Due to space constraints, we are unable to list the efficiency values for all input–output indicators. Therefore, we report the efficiency values of the key indicators in two stages.

**Table 8:** Efficiency of important input–output indicator.

Cluster	Year	CA	ABE	FSA	ISA	AGDP	RRDI	MSA
South	2016	0.906	1.000	0.934	0.872	1.000	0.943	1.000
	2017	0.881	0.927	0.941	0.885	0.993	0.968	1.000
	2018	0.873	0.931	0.924	0.845	0.975	0.979	0.985
	2019	0.875	0.918	0.956	0.868	0.961	0.989	0.986
	2020	0.871	0.906	0.905	0.833	0.930	0.961	0.980
North	2016	0.947	0.911	0.901	0.888	1.000	0.886	1.000
	2017	0.899	0.878	0.884	0.857	1.000	0.912	1.000
	2018	0.855	0.875	0.866	0.839	0.975	0.949	0.991
	2019	0.894	0.880	0.859	0.855	0.967	0.975	0.985
	2020	0.926	0.873	0.886	0.869	0.965	0.971	0.979

#### Input indicators

(1) Crop sown area (CA). The CA efficiency in the northern provinces is higher than that in the southern provinces. In terms of agricultural resources, climatic conditions, terrain topography, and agricultural mechanization conditions, the comparative advantage of the north is more obvious, which increases efficiency. The CA efficiency of the north reached a peak of 0.906 in 2016; the lowest efficiency year was in 2018, with an efficiency of 0.855, while the other three years were around 0.9, and from the trend of evolution showed a U-shaped inter-annual fluctuation of decreasing then increasing Trend. The CA efficiency value in the South, on the other hand, after reaching a peak of 0.906 in 2016, shows a year-on-year decreasing trend, with more room for improvement. This is also consistent with previous research, which found that CA efficiency in the southern provinces during the agricultural production stage is affected by multiple factors such as plot geometry, operational behavior, cropping patterns, and the level of agricultural mechanization. Unlike the cropping pattern of large farms in northern China, the small-plot compact cropping pattern is typical of agriculture in southern China, which is the reason for the low CA efficiency.

(2) Agricultural business entity (ABE). The overall efficiency of the North and South is above 0.8 every year, in which the ABE efficiency of the South performs better than that of the South, exceeding 0.9 in four years and 1 in one year. The ABE efficiency of the North performs slightly worse, exceeding 0.9 in only one year and exceeding 0.8 in another four years. Considering the gap between the North and the South, in the early stage of China’s reform and opening-up, the North was the centre of China’s manufacturing industry, and the development of the Agricultural business entity development relied on factors and investment to drive ahead of the development of the South, but led to the insufficient endogenous impetus for market-oriented reforms in the North, and the South relied on shipping and Yangtze River inland navigation to rise rapidly through market mechanism innovation. As the market in the south is more perfect, the marketisation of agricultural products is also easier to form a scale compared to the north Agricultural business entity’s agglomeration effect has a huge impact on the agricultural economy. For example, the marketization of agricultural products can promote China’s agricultural market resources more optimized, thus playing a role in reducing costs, and then increasing the disposable income of farmers, farmers realize the benefits of agriculture and will be more willing to enter the labor market, can bring more and more inexpensive manpower costs for the Agricultural business entity, thus forming a virtuous circle. In turn, the efficiency of ABE is better in the South than in the North.

(3) Financial support for agriculture (FSA). Financial support for agriculture in general public budget expenditures reflects the actual financial resources at the disposal of local governments in China after receiving transfer payments from the central government, which can truly map the differences in financial supply capacity among localities and the degree of importance attached to the industry. From the results of descriptive statistics, the North has more financial resources in the initial allocation than the South. Then, the FSA efficiency in the secondary allocation needs to be improved, and there is a tendency to expand between the northern and southern regions. The FSA efficiency of the southern provinces is consistently greater than 0.9, with the highest value of 0.956 in 2019. the lowest value of 0.905 in 2020. while the highest FSA efficiency of the southern provinces was in 2016, with an efficiency of 0.901. the performance of the rest of the years is stable between 0.878 and 0.880, with a large room for improvement.

(4) Infrastructure for sustainable agricultural development (ISA). The difference between the southern region and the northern region is not obvious, and the performance results show that the lowest value of ISA efficiency in the southern region appeared in 2020 at 0.833, and the highest value appeared in 2017 at 0.885. The rest of the years fluctuated between high and low values, which indicates that the ISA efficiency is highly unstable, and there is room for improvement. In the northern region, the lowest value of ISA efficiency appeared in 2018 as 0.839, and the highest value appeared in 2016 as 0.888, showing a U-shaped curve trend of decreasing and then increasing, but there is also a high room for improvement.

#### Output indicators

(1) Agricultural GDP (AGDP). Both regions have efficiency values greater than 0.9 each year, with the Northern region performing better, with AGDP efficiency greater than 0.960 each year and reaching the optimal production frontier in two years, with an efficiency value of 1. However, there is also a downward trend, from 1 in 2016 and 2017 to 0.965 in 2020. This may be due to the increase in costs due to the NKP outbreak and the intense competition in the marketplace, which thereby reduces AGDP efficiency. The Southern region also performed better, with AGDP efficiencies greater than 0.930 each year and 1 year with an efficiency value of 1. However, what raises concern is that the Southern region’s AGDP efficiency performance shows a trend of incremental decline. This may be related to the fact that the coastal cities of Guangxi, Zhejiang, Fujian, and Jiangsu in the southern region are subject to more frequent natural disaster events. Mainland China is located low in the mid-latitudes, with the Pacific Ocean to the east and the world’s highest terrain, the Tibetan Plateau, to the west, and the feedback relationship formed by the land and sea atmospheric systems. The strength of the polar high and the subtropical high is directly related to the degree of influence of the winter and summer winds. The increase in water temperature in the Pacific Ocean and the role of the El Niño phenomenon on atmospheric circulation and the source of storms have led to numerous and frequent climatic and oceanic disasters in China in recent years, which have seriously affected the performance of China’s AGDP efficiency.

(2) Per capita disposable income of rural residents (RRDI). The performance of RRDI efficiency is positive. Except for 2020, the RRDI efficiency in both the Southern and Northern regions maintained an upward trend. The RRDI efficiency of the Southern region increased from 0.943 in 2016 to 0.989 in 2019, but the efficiency score in 2020 fell back to the 2017 level, which needs to be taken into account by the relevant authorities. In contrast, the RRDI efficiency in the northern region grew from 0.886 in 2016 to 0.965 in 2019, although it does not perform as well as the southern region in terms of specific efficiency scores. However, the northern region is higher than the southern region in terms of incremental performance and performs better in 2020, which reflects the greater resilience of RRDI efficiency in the northern region. However, some effective measures need to be taken to stabilize RRDI efficiency and stop its decline.

(3) Number of rural residents guaranteed minimum subsistence allowance (MSA). The performance of MSA shows that it is the most efficient of all the output indicators, and the efficiency of MSA in both the South and the North is very good. The annual efficiency of the South region in 2016–2020 is all above 0.970, with the highest efficiency of 1.000 and the lowest efficiency of 0.980. And the annual average efficiency of the North region is also above 0.970, with the highest efficiency of 1.000 and the lowest efficiency shows that the living conditions of the disadvantaged groups in rural areas have been significantly improved, which is also consistent with the fact that China’s decision on winning the battle against poverty, considered by the Political Bureau of the Central Committee of the Communist Party of China (CPC) in 2015, will result in a major historic achievement in China’s fight against poverty by 2020.

### Causal network diagram analysis

In order to investigate the causal relationship between rainfall, poverty reduction efficiency in agricultural production and region, this paper uses Pearl^63^, who combined graph theory with Bayesian probability formulas, to propose the concept of Bayesian networks to analyze the causal relationship between the variables. Bayesian network (BN), is a directed acyclic graph that represents causal relationships between variables. The nodes represent the probability distributions of the variables, the directed edges between the nodes represent the causal relationships characterized by conditional probabilities, and the nodes are connected into a mesh by using the Bayesian conditional probability formula. The conditional probability distributions of the network nodes are obtained through prior knowledge and observation data, and the Bayesian network can calculate the posterior conditional probability distributions of other nodes to realize prediction or causal inference. Its expression is:

22 $$\begin{array}{*{20}c} {R_{{{\text{BN}}}} = \left\langle {X_{i} ,F} \right\rangle } \\ \end{array}$$

where $R_{{{\text{BN}}}}$ denotes the Bayesian network structure; $F$ is the set of directed edges; and $X_{i}$ is the set of all nodes in the network.

It is known that the Bayesian network structure contains the conditional independence assumption, i.e., under the condition that the parent node is known, each node is independent of the nodes that are not its descendants, and the conditional independence assumption expression is:

23 $$\begin{array}{*{20}c} {P\left( {X_{i} |X_{j} ,X_{n} } \right) = P\left( {X_{i} |X_{j} } \right)} \\ \end{array}$$

where $X_{i}$ denotes the parent node of $X_{j}$; P ($X_{i}$ ) represents the probability of the event occurring in the parent node; and $X_{n}$ represents the set of non $X_{i}$ child nodes.

For the study of Bayesian network models, there are usually two methods, structure learning and parameter learning. Structure learning refers to the process of using specific algorithms to extract the internal topology between variables and optimize the local structure of the network with the help of known data and prior knowledge. Parameter learning is the process of determining the conditional probabilities of different nodes of a Bayesian network as well as specific parameters based on actual data as well as the experience of professionals. Rainfall, agricultural production and government poverty reduction is a system with a complex interaction mechanism, and the same evaluated unit may face different risks in different time and space, so it is extremely difficult to perform data statistics directly, which cannot meet the data set needed for Bayesian structured network learning and parameter learning. Therefore, this study mainly utilizes the way of parameter learning based on actual data to confirm the occurrence probabilities of each node P(${X}_{i}$), P(${Y}_{i}$) and P(${Z}_{i}$). Since the nodes studied in this paper are all binary events, the probability that the studied node does not occur can be determined when the occurrence probability of the studied node is determined, as explained in Table 9:

**Table 9:** Interpretation of Jointly Distributed Probability Variables.

	X	Y	Z
0	North	Unimproved	No Rain
1	South	Improved	Rain

Where P(X = 1) denotes South, P(Y = 1) denotes Improved and P(Z = 1) denotes the effect of rainfall is considered. According to the results of parameter learning based on real data can be derived:

1. P (Y = 1|X = 1,Z = 1) = 0.1012
2. P (Y = 1|X = 1,Z = 0) = 0.0160
3. P (Y = 1|X = 0,Z = 1) = 0.1765
4. P (Y = 1|X = 0,Z = 0) = 0.0062

**Figure 6:**
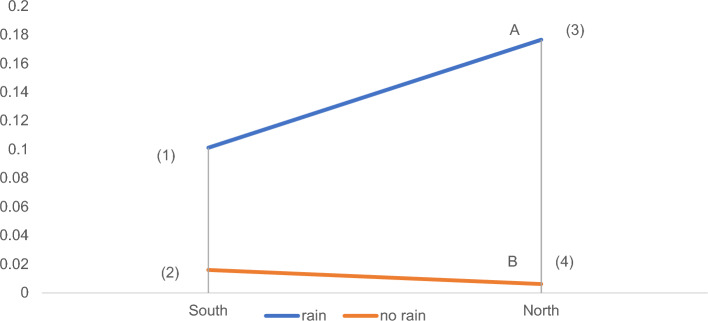
Convex combination.

According to the above calculation process, we get the a priori probabilities and conditional probabilities of the variables, and substitute the probabilities of all the nodes into the convex combination of Fig. 6, where A is a point that is free to move between the line segments (1) and (3), and B is a point that is free to move between the line segments (2) and (4), we can get the four statements.

- Statement 1
  - Point A > Point B, For Statement 1 to hold, it is implicitly necessary that point A be as close as possible to point (3), which means that the northern region has a higher probability of using rainfall as an exogenous variable compared to the southern region. That is: region → rainfall.
- Statement 2
  - Point (3) > Point (4), the implication of which is that the northern region has a higher probability of improvement after considering rainfall than without considering rainfall. That is: region → rainfall → efficiency improvement.
- Statement 3
  - Point (2) > Point (4), the implication of which is that the southern region has a higher probability of efficiency improvement than the northern region if rainfall is ignored. That is: region → efficiency improvement.
- Statement 4
  - Point (3) > Point (1), the implication of which is that if rainfall is taken into account, the probability of efficiency improvement is higher in the Northern region than in the Southern region. I.e.: region → efficiency improvement.

**Figure 7:**
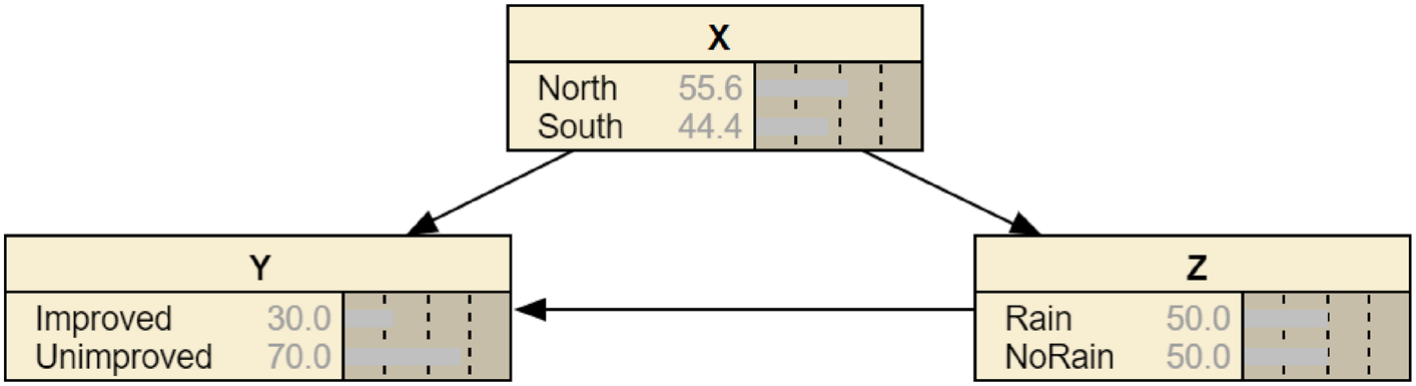
Bayesian network for area, rainfall and efficiency improvements after calculating conditional probability tables (2016–2020).

Finally we used Netica 7.01 free version software (https://norsys.com/download.html.) to complete the Bayesian network visualization and the results are shown in Fig. 7:

## Conclusions and policy recommendation

### Empirical analysis conclusions

In this paper, using rainfall as a proxy variable for the atmospheric environmental system, the dynamic DDF model of the common boundary network under the introduction of exogenous DEA was used to measure the agricultural production efficiency and government poverty reduction efficiency of 27 provinces in China from 2016 to 2020, and the dynamic changes in the efficiency of the agricultural production stage and the government poverty reduction stage in the southern region and the northern region of China were discussed, and the results of the study showed that:(1) rainfall as an exogenous variable has a significant effect on agricultural production efficiency. When rainfall is taken into account, the overall efficiency increases in both the southern and northern regions, suggesting that changes in rainfall indirectly reflect the impact of the climate environment on sustainable agricultural development. This provides a valuable reference for future agricultural planning and water management, emphasizing the importance of considering meteorological factors when assessing agricultural efficiency. (2) The relatively lower efficiency of government poverty reduction in the northern provinces compared to the southern provinces is associated with more frequent natural disaster events. This suggests that rainfall not only affects agricultural production but also has a significant impact on the efficiency of the government’s poverty reduction phase. In contrast, the U-curve trend in government poverty reduction efficiency in the southern provinces may be affected by the frequency of natural disasters. There is a need to focus on the root causes of the economic gap between the North and the South and formulate appropriate policies to enhance the efficiency of poverty reduction. (3) The study also reveals that there are differences in market-based reforms between the South and the North, with different levels of intervention in factor markets. The government of the southern region pays more attention to the principle of fair competition in the market, while the government of the northern region is more inclined to adopt preferential policies and subsidy strategies, which leads to a relative lag in the market-based reform process in the northern plate, thus affecting the government’s efficiency in poverty reduction. This signifies that more attention should be paid to the development and operation of the market mechanism in poverty reduction policy formulation to improve the government’s efficiency in poverty reduction.

### Policy recommendations

Combined with the findings of this paper, the following policy implications can be drawn: (1) Enhancing Agricultural Production Efficiency and Sustainability. To improve agricultural production efficiency and sustainability, it is recommended to establish a nationwide meteorological information system. This system should monitor and predict rainfall patterns, facilitating the development of a responsive agricultural planning framework. Additionally, workshops for farmers on climate-smart agricultural practices should be organized to ensure adaptive and informed farming methods. (2) Improving Disaster Resilience in Northern Provinces. Addressing the relatively lower efficiency of government poverty reduction in northern provinces, particularly due to frequent natural disasters, requires the creation of a dedicated fund for disaster-resilient infrastructure. This fund should support initiatives such as early warning systems, community-based disaster preparedness training, and the construction of resilient shelters. Regular drills and simulations should also be conducted to enhance community response during natural disasters. (3) Rectifying Disparities in Market-Based Reforms. To rectify the disparities in market-based reforms between the Southern and Northern regions, a comprehensive review of market intervention policies in the northern provinces is recommended. Incentives should be provided to businesses adhering to fair competition principles, and training programs for government officials on market-oriented strategies and fair competition principles should be developed. (4) Promoting Fair Competition in Factor Markets. Promoting fair competition in factor markets in the northern region can be achieved through awareness campaigns and guidance to businesses on ethical market practices. The creation of a certification system for businesses adhering to fair competition principles, along with collaboration between the government and industry associations, can further ensure the enforcement of fair competition. (5) Efficiency in Poverty Reduction. Finally, to improve the efficiency of poverty reduction, it is crucial to establish a task force dedicated to studying and recommending improvements to market mechanisms in poverty reduction policies. Pilot projects in the northern provinces should focus on market-driven approaches, with subsidies aligned with fair competition principles. Regular assessments and adjustments to policies based on the performance of these pilot programs are essential for continual improvement.

## Data Availability

Data is provided within the manuscript.
